# Reproductive Toxicity and Recovery Associated With 4-Non-ylphenol Exposure in Juvenile African Catfish (*Clarias garepinus*)

**DOI:** 10.3389/fphys.2022.851031

**Published:** 2022-04-11

**Authors:** Alaa El-Din H. Sayed, Zainab Eid, Usama M. Mahmoud, Jae-Seong Lee, Imam A. A. Mekkawy

**Affiliations:** ^1^Department of Zoology, Faculty of Science, Assiut University, Assiut, Egypt; ^2^Department of Biological Sciences, College of Science, Sungkyunkwan University, Suwon, South Korea

**Keywords:** E2, catfish, FSH, eryptosis, hemotoxicity, erythrocytes

## Abstract

Although, the effects of 4-non-ylphenol (4-NP) on fish’s reproductive hormones were assessed in several studies using adult models, however, the effect of this endocrine disruptor on immature fish’s reproductive hormones was not addressed commonly. We aimed to study the apoptosis induction, hematotoxicity, reproductive toxicity, and the recovery associated with 4-NP exposure in juvenile African catfish [*Clarias garepinus*) using some hormones [17β-estradiol (E2), testosterone (T), follicle-stimulating hormone (FSH), and luteinizing hormone (LH)] and gonad histology as biomarkers. The toxic effects of 4-NP have been studied in many animal models, but there is still limited knowledge about the dose-dependent damage caused by 4-NP exposure in juvenile *Clarias gariepinus*. A healthy juvenile *C. gariepinus* was categorized into four groups (*n* = 3/group; three replicates in each group). The first group was the control, and the other three groups were subjected to 4-NP concentrations as 0.1, 0.2, and 0.3 mg/L for 15 days; they were left for a recovery period of another 15 days. The reproductive hormones of *C. gariepinus* exposed to 4-NP for 15 days exhibited significant variations between the treatment groups and the control (*P* < 0.05), which were evident in E2 and *T*-values, whereas FSH, LH, total protein, and lipid peroxidation values showed non-significant differences among all groups at *P* < 0.05. Such a situation referred to the fact that the 15-day recovery period was insufficient to remove the impacts of 4-NP doses in concern. The trend of dose-dependent increase/decrease was recorded for T, E2, FSH, and LH. The histopathological alterations of 4-NP treated in gonad tissues were recorded in juvenile *C. gariepinus*, reflecting their sensitivity to 4-NP estrogenic-like effects. Overall, our results investigate that recovery has improved the reproductive toxicity caused by 4-NP in juvenile *C. garepinus.* Significant variations between the treated groups and the control group (*P* < 0.05) were evident in hematological parameters except for hemoglobin (Hb), mean corpuscular volume (MCV), mean corpuscular hemoglobin (MCH), and mean corpuscular hemoglobin concentration (MCHC). The parameters exhibiting significance decreased with such increased doses [red blood cells (RBCs), hematocrit (Hct), and white blood cells (WBCs)]. Similar patterns of significant variations toward the increase or decrease were recorded following the 15-day recovery period. Apoptotic frequency in erythrocytes and brain cells has increased significantly with increased 4-NP exposure, indicating that 4-NP caused cytotoxic effects, such as apoptosis in a dose-dependent manner. However, these cellular alterations greatly decreased after the 15-day recovery period.

## Introduction

Water pollution is considered one of the biggest problems worldwide, especially in Egypt (El-Kowrany et al., 2016; Mekkawy et al., 2019). It is exacerbated by the fields of ecology, agriculture, veterinary science, pharmacology, and medicine (Khandale et al., 2018). Multiple toxicological studies have identified industrial phenolic waste as the most common water pollutant (Buikema et al., 1979; Araujo et al., 2018). Recently, chemical pollution with endocrine-disrupting chemicals (EDCs) is considered a serious international issue around the world. The EDCs were identified as exogenous substances that alter the endocrine system causing negative effects on the health of an intact organism (Rattan et al., 2018). In the past two decades, EDCs caused an imbalance in the reproductive hormones of organisms (Shirdel et al., 2020). The 4-NP is one of the EDCs and takes the attention of many investigators as it has estrogenic effects, including developmental and physiological abnormalities on reproductive impairments (Sayed et al., 2012a). The 4-NP is strongly lipophilic (Baatrup and Junge, 2001), accumulates in the lipids and cell membranes of the organism, and can enhance estrogen-sensitive processes (Petit et al., 1999) affecting the reproductive system of male fish (Zade et al., 2018).

Shirdel et al. (2020) reported that, in the Caspian Sea environment, the raised concentrations of 4-NP could affect the aquatic animals residing in the Caspian Sea, as they could impair reproductive hormonal balances and gonad development. The use of 4-NP has been restricted by European Union since 2003 (European Parliament of the Council, 2006) due to its disruptive effects that target the endocrine system and alter its functions in several ways. Although, given these restrictions, the environment is still containing NPs and nonylphenol ethoxylates (NPEOs) because the imported products contain these unbanned substances (Ruczyńska et al., 2020) that are still widely accessible in commercial detergents in most of the countries where the production and use of these compounds are still not regulated by laws (Klosterhaus et al., 2013).

According to Choi et al. (2010), exogenous environmental cues like photoperiod and temperature, and endogenous hormonal cues through the neuroendocrine brain-pituitary-gonad (BPG) axis are considered the main components that play important roles in regulating the complex reproduction process in fish. As the endocrine system is sophisticated, so is the xenobiotics’ mechanism; the dysfunction it causes is highly complicated and not yet fully comprehensible (Ruczyńska et al., 2020).

Sex hormones, for example, follicle-stimulating hormone (FSH), luteinizing hormone (LH), and 17β-estradiol (E2) are synthesized, released, and regulated *via* gonadotropin-releasing hormone (GnRH) (Yadetie and Male, 2002). Thus, it is considered the master regulator of puberty and the adult reproductive cycle (Zohar et al., 2010). Estradiol 17-b (E2) is responsible for controlling the neuroendocrine feedback of the reproductive cycle in the brain-pituitary-gonadal axis (Yadetie et al., 1999). Yadetie and Male (2002) reported that regulating gonadal development’s steroidogenesis and ovulation is carried out *via* pituitary gonadotropins’ FSH (GTH-I), and LH (GTH-II), which are considered as key reproductive hormones. The FSH plays a vital role in mediating vitellogenesis and spermatogenesis, whereas LH emboldens the final maturation of oocytes and termination in fish (Choi et al., 2010). This compound affects the male and female reproductive systems causing different dysfunctions, altering the levels of LH and FSH (Sayed et al., 2012a), and may lead to disturbance in the development of primary and secondary sexual characteristics (Partridge and Jones, 2010).

Different blood cell indices, including hemotoxicity and biochemistry, are used as major biomarkers of systemic responses to environmental stress in fish (Mekkawy et al., 2011; Abou Khalil et al., 2017; Sayed and Soliman, 2018). In addition to apoptosis, micronucle and nuclear abnormalities, fish erythrocyte deformations have also been considered in toxicological studies on pollution-stress responses (Talapatra and Banerjee, 2007; Mekkawy et al., 2011; Sayed et al., 2016a,2019b). Because the brain and spinal cord play an essential role in fish physiology, the brain tissue is often studied in fish toxicology (Sayed et al., 2019b). It is postulated that brain biomarkers may be effective for pollution biomonitoring and may provide warning signals of pollutant exposure (Ferrando and Andreu-Moliner, 1991; Tabassum et al., 2015). Among these biomarkers, apoptosis is considered to be one of the most frequent mechanisms of neuronal cell death in several pathological alterations, especially the chronic degenerative brain weaknesses (Chi et al., 2018). Apoptosis is related to signs of irregular cell morphology, such as morphological alterations, nuclear abnormalities, and DNA damage (Murakawa et al., 2001; Cavas et al., 2005; Talapatra and Banerjee, 2007; Sayed et al., 2016a).

The African sharptooth catfish, *Clarias gariepinus*, is distributed throughout Africa and has significant economic importance (Nguyen and Janssen, 2002). Recently, this species has been used as an excellent model for toxicological studies (Mekkawy et al., 2011, 2019; Kotb et al., 2018).

Various studies on the effects of 4-NP on the fish reproductive hormones were analyzed, but most of these studies have been done on adult fish (Sayed and Ismail, 2017), and according to Shirdel et al. (2020), the effect of this substance is less detected on the reproductive hormones of immature fish. Although there are many studies on 4-NP in fish, more research must be done on its effects as an estrogenic and toxic pollutant, and whether it is a dose-dependent or chemical-like hormone. Consequently, the main objective is to study the reproductive toxicity and recovery associated with 4-NP exposure in male and female juvenile African catfish (*Clarias garepinus*) and to investigate dose-dependent damage caused by 4-NP using hematobiochemical parameters, gonadal histopathological alterations, and apoptosis in erythrocytes and brain tissue as biomarkers.

## Materials and Methods

### Specimens’ Collection

A private farm in Assiut provided healthy juvenile *C. gariepinus* of both sexes with a mean weight of 108.18 ± 3.5 g and a mean length of 26 ± 0.3 cm. As part of the lab conditions adaption, the fish were maintained all together in 100-L rectangular tanks containing dechlorinated and ventilated tap water for 2 months. The fish were fed with a commercial pellet diet (SKRETTING Company, Egypt) containing 3% of their body weight per day, and the water was changed daily to remove contaminants from metabolic wastes. The African catfish were checked after acclimatization to ensure that they were free of external parasites and healthy (F.H.S., 2004).

### Experimental Design

Four groups of fish were created (*n* = 12/group; three replicates in each group). The first group served as the control, while the other three were exposed for 15 days to 4-NP at concentrations of 0.1, 0.2, and 0.3 mg/L, respectively, followed by 15 days of post-exposure (Mekkawy et al., 2011; Lee et al., 2018; Sayed and Soliman, 2018). Every day, half of the water was changed and redosing was done. Sigma–Aldrich provided the 4-NP (analytical standard 100%) (Tokyo, Japan). Experimental setup, guidelines, and fish handling were approved by the Research and Ethical Committee of the Faculty of Science, Assuit University, Assuit, Egypt.

### Blood Sample Collection, Hematological Parameters, and Reproductive Hormones Measurements

Fish from each group were randomly selected and anesthetized using ice at the end of the 15-day exposure and 15-day post-exposure periods to reduce stress (Wilson et al., 2009). Blood was obtained from the caudal veins and transferred into heparinized tubes for hematological measurement, and blood smears were placed on clean glass slides in triplicate for each fish (i.e., nine slides from each group). The counting of the RBCs and WBCs was performed using a hemocytometer and light microscope (Stevens, 1997). The microhematocrit method of Hesser (1960) was used to detect the Hct. The Hb values were assessed according to the methods of Lee et al. (1999). The RBC indices were calculated from the RBC counts, Hb, and Hct according to the formulas suggested by Lee et al. (1999). Some of the blood was taken in non-heparinized, clean and dry tubes, was allowed to clot at room temperature, and then centrifuged at 5,000 rpm, at 4°C for 20 min, and the serums were separated to measure the reproductive hormones, such as E2, T, FSH, and LH, using the ELISA kits (Diagnostics Biochem Canada Inc., 1020 Hargrieve Road, London, Ontario N6E 1P5, Canada). Estradiol and testosterone were assessed using a kit with Cat. Nos.CANE-1430 and CAN-TE-250, respectively, and according to the method of Check et al. (1995). The FSH was assessed to the method of Knobil (1980) using a kit (Cat.No.CANFSH-4060). The LH was assessed according to the method of Cumming et al. (1985) using a kit (Cat. No. CAN-LH-4040).

### Erythrocyte’s Morphological Alterations and Nuclear Abnormalities

According to the protocols of Hamed et al. (2019), the blood smears were preserved in absolute methanol, air dried, and stained with 6% Giemsa stain. Under a 40x objective with a 10x eyepiece Omax microscope with 14 MP USB Digital Camera, slides were selected, coded, randomized, and assessed by one person based on staining quality as stated by Schmid (1975) and Al-Sabti and Metcalfe (1995) (CS-M837ZFLR-C140U; A35140U3; China).

### Apoptosis in Erythrocytes and Brain Tissue (Telencephalon)

An acridine orange staining (Cat. No. A1031, Life Technologies, Carlsbad, CA, United States) was used for apoptosis detection. After xylene deparaffinization of wax from 7 μm sections of the brain, a descending series of alcohols, and then water, a modified protocol (Sayed et al., 2016a), was used to detect apoptosis in the RBCs and brain tissue. Apoptotic cells were examined using a fluorescence microscope (BX50; Olympus, Japan), with a built-in digital color video camera (DP-70; Olympus, Japan).

### Residue of 4-NP in Brain Tissue

Brain tissue was harvested after a 4-NP treatment and after the recovery period and kept at −80°C. The 0.1 g samples were processed with methanol for 4-NP extraction following the protocol of Gautam et al. (2015) to determine the accumulation of 4-NP in brain tissue.

### Quantitative Histological Alterations

After 15 days of exposure and 15 days afterward, fish from each group were selected at random and anesthetized with ice to reduce stress (Wilson et al., 2009). The gonads were taken from the fish, which were then divided into minute pieces and fixed in Davidson fixative for 24 h. They were dehydrated and then imbedded in paraffin wax using a graded series of ethanol. Blocks were sectioned with a microtome at a thickness of 7 microns. The sections were stained with Harris’s hematoxylin and Eosin stain (H&E) (Bancroft and Stevens, 1982) to assess the usual histopathology. According to Sayed and Authman (2018), the percentage of atresia in the ovary and the number of Sertoli cells in the testes were measured (2014). Finally, photos were acquired using an Omax microscope with a 14 MP USB Digital Camera and a 40 objective with a 10 eyepiece (CS-M837ZFLR-C140U).

### Statistical Analysis

Basic statistics, such as means, standard deviations, and ranges, were calculated. One-way ANOVA was used to investigate the pattern of variation at 0.05 and 0.001 significance levels, using the SPSS software (IBM Corp, 2012). For multiple comparisons, the Tukey-HSD test and Dunnett *t*-tests were used.

## Results

### Serum Sex Steroids, Gonadotropins, and Hematological Parameters

The reproductive hormones of *C. gariepinus* exposed to 4-NP for 15 days were recorded in Table 1; it shows that significant variances between the treatment groups and the control (*P* < 0.05) were evident in estradiol and testosterone values. However, a non-significant lower testosterone was observed in the group exposed to 0.1 mg/L 4-NP compared to the control group, whereas FSH, LH, total protein, and lipid peroxidation values showed a non-significant difference among all groups at *P* < 0.05, except in total protein, the group exposed to 0.1 mg/L 4-NP and, in lipid peroxidation, the group exposed to 0.3 mg/L 4-NP showed significant difference compared to the control group (*P* < 0.05). The measures that show significant differences between treatments tend to increase (LH, estradiol, and lipid peroxidation) or decrease (LH, estradiol, and lipid peroxidation) as the 4-NP doses increase from 0.0 to 0.3 mg/L (FSH, testosterone, and total protein). After 15 days, similar patterns of significance and trend deviations toward increase or decrease were seen (Table 1). In the fact, the 15-day postexposure period was insufficient to erase the effects of the 4-NP doses. T, estrogen, FSH, and LH all showed a dose-dependent increase/decrease pattern.

**TABLE 1:** Effect of different doses of 4-non-ylphenol (4-NP) on hormones, total protein, and lipid peroxidation mean ± SE (range) in juvenile African catfish (*Clarias gariepinus*) exposed to 0.1, 0.2, and 0.3 mg/L4-NP for 15 days and recovery for 15 days.

Treatments parameters	Control		4-NP As (0.1 mg/L)		4-NP As (0.2 mg/L)		4-NP As (0.3 mg/L)	
	Exposure	Recovery	Exposure	Recovery	Exposure	Recovery	Exposure	Recovery
FSH (U/L)	0.14 ± 0.012^a^ (0.12–0.16)	0.163 ± 0.003^a^ (0.16–0.17)	0.113 ± 0.01^ab^ (0.1–0.13)	0.143 ± 0.003^b^ (0.14–0.15)	0.103 ± 0.01^ab^ (0.09–0.12)	0.123 ± 0.0033^c^ (0.12–0.13)	0.087 ± 0.012^b^ (0.06–0.1)	0.1067 ± 0.003^d^ (0.1–0.11)
LH (U/L)	0.235 ± 0.01^a^ (0.23–0.25)	1.05 ± 0.8^a^ (0.24–2.65)	0.259 ± 0.01^a^ (0.24–0.27)	0.256 ± 0.003^a^ (0.25–0.26)	0.263 ± 0.001^a^ (0.24–0.28)	0.277 ± 0.0012^a^ (0.28–0.28)	0.294 ± 0.0027^b^ (0.29–0.3)	0.287 ± 0.001^a^ (0.28–0.28)
T (pg/ml)	2.07 ± 0.035^a^ (2–2.11)	2.2 ± 0.058^a^ (2.1–2.3)	1.99 ± 0.058^a^ (1.9–2.1)	2.067 ± 0.03^a^ (1.08–1.1)	1.83 ± 0.027^b^ (1.78 –1.87)	1.99 ± 0.061^ab^ (1.89–2.1)	1.596 ± 0.0267^c^ (1. 57–1.65)	1.797 ± 0.074^b^ (1.65–1.89)
E2 (pg/ml)	1.08 ± 0.023^a^ (1.04–1.12)	1.023 ± 0.01^a^ (1.01–1.04)	1.223 ± 0.003^b^ (1.22–1.23)	1.09 ± 0.006^a^ (1.08–1.1)	1.307 ± 0.028^b^ (1.25–1.34)	1.13 ± 0.035^a^ (1.09–1.2)	1.397 ± 0.012^c^ (1.38–1.42)	1.247 ± 0.0467^b^ (1.2–1.34)
TP (nmol/mg tissue)	10 ± 0.360^a^ (9.43–10.67)	7.08 ± 1.23^a^ (4.86–9.05)	4.63 ± 0.084^b^ (4.48–4.76)	5.43 ± 0.22^a^ (5.05–5.81)	7.27 ± 0.498^a^ (6.29–7.9)	7.11 ± 0.825^a^ (5.71–8.57)	8.48 ± 1.12^a^ (6.38–10.19)	3.9 ± 0.33^a^ (3.24–4.29)
LPO (nmol/mg tissue)	310.58 ± 7.34^a^ (298.75–324.02)	212.18 ± 35.78^a^ (145.16–267.43)	174.61 ± 5.49^a^ (164.67–183.62)	206.93 ± 8.13^a^ (192.16–220.21)	350.9 ± 24.023^a^ (304.1–383.72)	341.02 ± 40.14^a^ (274.58–413.28)	668.82 ± 29.47^b^ (504–811.57)	304.71 ± 25.68^a^ (253.64–335)

*Significant differences from the control group values were accepted at P < 0.05. n = 3. Values with the same letters within a parameter are not significantly different at.05 level (horizontal comparison). FSH, Follicle-stimulating hormone; LH, Luteinizing hormone; T, Testosterone; E2, Estradiol; TP, Total protein; LPO, Lipid peroxidation, NP, 4-non-ylphenol.*

The hematological parameters of *C. gariepinus* exposed to 4-NP for 15 days were recorded in Table 2. The significant differences (*P* < 0.05) between the treatment groups and the control group were evident in all parameters, except for Hb, mean corpuscular volume (MCV), mean corpuscular hemoglobin (MCH), and mean corpuscular hemoglobin concentration (MCHC). The RBCs, Hct, and WBCs significantly decreased with increasing 4-NP doses from 0.0 in the control to 0.3 mg/L. Similar changes were recorded after the 15-day recovery period (Table 2), indicating that 15 days was insufficient to allow the catfish to recover from 4-NP exposure.

**TABLE 2:** Effect of different doses of 4-NP on the hematological parameters mean ± SE (range) of the African catfish *C. gariepinus* after exposure to 4-NP for 15 days and recovery for 15 days (*n* = 3).

Treatments parameters	Control		0.1 mg/L 4-NP		0.2 mg/L 4-NP		0.3 mg/L 4-NP	
	Exposure	Recovery	Exposure	Recovery	Exposure	Recovery	Exposure	Recovery
RBC’s ×	3.15 ± 0.02^a^	3.175 ± 0.062^a^	2.975 ± 0.075^ab^	3.025 ± 0.1^ab^	2.8 ± 0.04^b^	2.825 ± 0.04^bc^	2.575 ± 0.047^c^	2.76 ± 0.02^c^
(10^6^/mm^3^)	(3.1–3.2)	(3–3.3)	(2.8–3.1)	(2.9–3.2)	(2.7–2.9)	(2.7–2.9)	(2.5–2.7)	(2.7–2.8)
Hb (Mg/dl)	9.62 ± 0.54^a^	10.32 ± 0.160^a^	9.665 ± 0.11^a^	9.995 ± 0.09^a^	8.99 ± 0.23^a^	9.4 ± 0.24^b^	8.475 ± 0.125^a^	8.67 ± 0.10^c^
	(7.98–10.2)	(10.14–10.8)	(9.4–9.9)	(9.78–10.2)	(8.4–9.5)	(8.8–9.8)	(8.2–8.8)	(98.5–8.9)
Hct (%)	37 ± 0.6^a^	37.25 ± 0.48^a^	36.375 ± 0.3^a^	35.75 ± 0.54^b^	34.81 ± 0.29^b^	34.35 ± 0.29^bc^	33.17 ± 0.25^c^	33.34 ± 0.23^c^
	(35.7–38.5)	(36.4–38.5)	(35.5–36.8)	(34.5–36.8)	(34.5–35.7)	(33.5–34.9)	(32.5–33.7)	(32.6–33.72)
MCV (mm^3^)	117.48 ± 2.1^a^	117.40 ± 1.70^a^	122.53 ± 3.57^a^	118.38 ± 3.24^a^	124.41 ± 2.07^a^	121.73 ± 2.9^a^	129 ± 3.04^a^	120.73 ± 1.6^a^
	(111.56–120.64)	(113.75–122)	(114.51–130)	(110–125.8)	(118.96 –127.8)	(115.5–129.2)	(122.6–134.8)	(116.6–124.2)
MCH (Pg)	30.57 ± 1.89^a^	32.54 ± 0.79^a^	32.54 ± 0.88^a^	33.10 ± 0.93^a^	32.18 ± 1.28^a^	33.27 ± 0.71^a^	32.93 ± 0.66^a^	31.40 ± 0.36^a^
	(24.93–32.9)	(30.72–34)	(30.83–35)	(30.5–34.8)	(28.96–35.18)	(31.7–35)	(31.11–34)	(30.3–32)
MCHC (g/dl)	26.07 ± 1.8^a^	27.71 ± 0.50^a^	26.58 ± 0.52^a^	27.96 ± 0.24^a^	25.84 ± 0.73^a^	27.37 ± 0.75^a^	25.55 ± 0.53^a^	26.02 ± 0.44^a^
	(20.72 –28.57)	(26.33–28.8)	(25.54–27.88)	(27.6–28.6)	(24.34–27.53)	(25.2–28.4)	(24.55–27.07)	(25.3–27.2)
WBC’s (cell/mm^3^)	11.4 ± 0.24^a^	11.45 ± 0.125^a^	11.002 ± 0.3^ab^	10.97 ± 0.29^a^	10.35 ± 0.06^bc^	10.72 ± 0.11^ab^	9.82 ± 0.26^c^	10.1 ± 0.14^b^
	(10.8–11.9)	(11.2–11.8)	(10.11–11.5)	(10.2–11.6)	(10.2–10.5)	(10.4–10.9)	(9.2–10.5)	(9.9–10.5)

*Significant differences between the control group and treatments.*

### Ovary Histopathology

**FIGURE 1:**
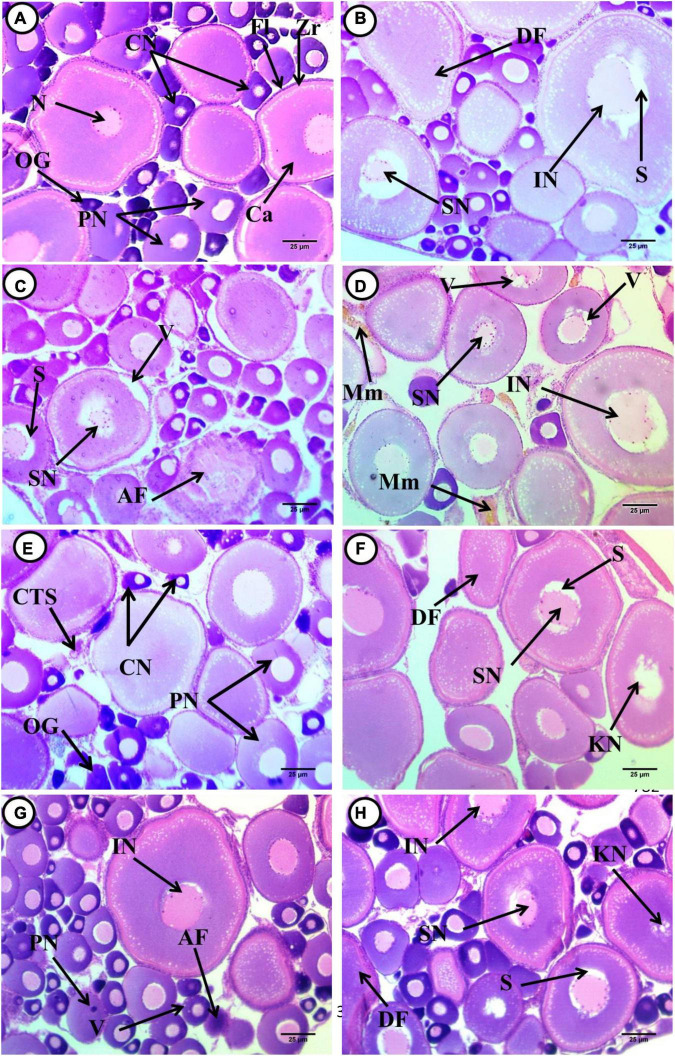
Transverse sections of juvenile African catfish (C. gariepinus) ovary. **(A)** Control fish after exposure to 4-non-ylphenol (4-NP), **(B)** fish exposed to 0.1 mg/L 4-NP, **(C)** fish exposed to 0.2 mg/L 4-NP, **(D)** fish exposed to 0.3 mg/L 4-NP, **(E)** control fish after recovery period, **(F)** fish exposed to 0.1 mg/L 4-NP dose after the recovery period, **(G)** fish exposed to 0.2 mg/L 4-NP dose after the recovery period, and **(H)** fish exposed to 0.3 mg/L 4-NP dose after the recovery period, Oogonia (OG), chromatin nucleolar stage (CN), and perinucleolar stage (PN). Primary envelope (zona radiata) (Zr), follicular layer (Fl), cortical alveoli (Ca), nucleus consist of more than four nucleoli (N), deformed shape of cortical alveoli stage (DF), irregular nucleus (IN), unstained space around the nucleus (S) shrunken nucleus (SN), the atretic follicle (AF), vacuoles (V) and shrunken nucleus (SN), vacuoles (V), irregular nucleus (IN) and Melanomacrophage centers (Mm), karyolitic nucleus (KN), and pyknotic nucleus (PN). (H&E ×400).

Transverse sections of the ovary of normal juvenile *C. gariepinus* in exposures and recoveries of both group were stained by H&E, showing the normal histological architecture of the ovary. The diversity of sizes from chromatin nucleolar stages, which consist of deeply stained cytoplasm with centrally located nuclei, the Perinucleolar stages with different sizes containing less basophilic cytoplasm, and the nuclei were located perinucleolarly and consisted of more than four nuclei. The cortical alveoli formation stage, or yolk vesicle stage, contained a central nucleus that had a large number of peripherally arranged nucleoli. Infolded nuclear membrane and nuclei were located as perinucleolar. A thin acidophilic primary envelope (zona radiata) was visible. The follicular layers consisting of simple cuboidal were surrounded with the stratified squamous thecal layer. Ovocoel containing connective tissue stroma was observed (Figures 1A,E). The sections of fish ovary exposed to 0.1 mg/L 4-NP, showing a large number of different-sized cortical alveoli formation stages, were observed, containing a huge faint basophilic cytoplasm with different stages of nuclei degeneration, separation of nuclei from their cytoplasm, and shrunken with irregularity in their rim surrounded by unstained space. An increase in cortical alveoli’s size and rows was observed at the periphery of the cytoplasm. Zona radiata was clearly visible and the follicular layers consisted of simple cuboid surrounded with stratified squamous thecal layer. The atretic stages and connective tissues’ stroma were increased and deeply stained pyknotic and eccentrically located nuclei were observed. The invaginated boundary of the perinucleolar stage was noticed, which lost its rounded shapes (Figure 1B).

In sections of fish ovary exposed to 0.2 mg/L 4-NP perinucleolar and cortical alveoli, the stage was dominant while a small number of chromatin stages was observed. The perinucleolar stage, which contains both eccentrically located nuclei and degenerated ones, was noticed (atretic follicle), and deformed shapes of the cortical alveoli stage, which appeared ovoid in shapes, shrunken nuclei surrounded by unstained space, and detachment of their outer boundaries of both stage and nuclei, were also observed (Figure 1C). A fish ovary exposed to 0.3 mg/L 4-NP, showing deformed shapes of the cortical alveoli formation stage, which appeared ovoid in shapes, and cytoplasm appeared less basophilic in staining and contained vacuoles. Also, thickening of outer follicular layer and invasion of cortical vesicles into the center of stage, beginning of yolk formation, deformation, and dislocation of nuclei into the periphery of cytoplasm, and irregularity of their rim and separated from nearby cytoplasm were observed. The malignant follicles (Atretic follicles) with irregular rim and detachment of their stratified squamous epithelium were observed. Cytoplasm appeared as heterogeneous and contains acidophilic materials, while the ovocoel contains the connective tissues’ storma, and the melanomacrophage centers appeared (Figure 1D).

After post-exposure, with fish exposed to 0.1 mg/L 4-NP, the dominant stages are the cortical alveoli formation, perinucleolar, and chromatin nucleolar. There was deformation in shapes of cortical alveoli with irregular outer rim containing faint basophilic cytoplasm, with shrunken and infolded nuclei, which were surrounded by unstained space that separated it from contact cytoplasm. Other stages with a karyolytic nucleus were observed. The perinucleolar is a second dominant stage, which appeared deformed in shapes, shrunken nuclei, left space from its surrounding cytoplasm; degenerated, while the other is with the pyknotic nucleus. A vacuolated stage was also observed. Few stages of chromatin nucleolar were observed with eccentric nuclei, and connective tissues stroma was observed in the ovocoel (Figure 1F). In sections of fish ovary exposed to 0.2 mg/L 4-NP post-exposure, there was an increase in the number of chromatin nucleolar and perinucleolar stages, and few cortical alveoli formations were observed. Chromatin nucleolar ones appeared with deeply basophilic cytoplasm, with eccentrically located nuclei. The diversity in size of perinucleolar stages with less basophilic cytoplasm and nuclei were slightly eccentric and pyknotic in this stage. Deformation in shapes and boundary of cortical alveoli stages, less basophilic and vacuolated cytoplasm, detachment of the nuclei from surrounding cytoplasm, and others that are shrunken were observed. Vacuoles’ invasion to the core of these stages was noticed at the beginning of yolk formation. The ovocoel contains connective tissue stroma (Figure 1G). In fish ovary exposed to 0.3 mg/L 4-NP after post-exposure, the dominant stages are the cortical alveoli formation, which appeared deformed in shapes with acidophilic cytoplasm. Many stages of nuclear degeneration were observed from atrophy or shrunken, had an infolded outer rim, and were dissociated ones (Figure 1H).

### Testis Histopathology

**FIGURE 2:**
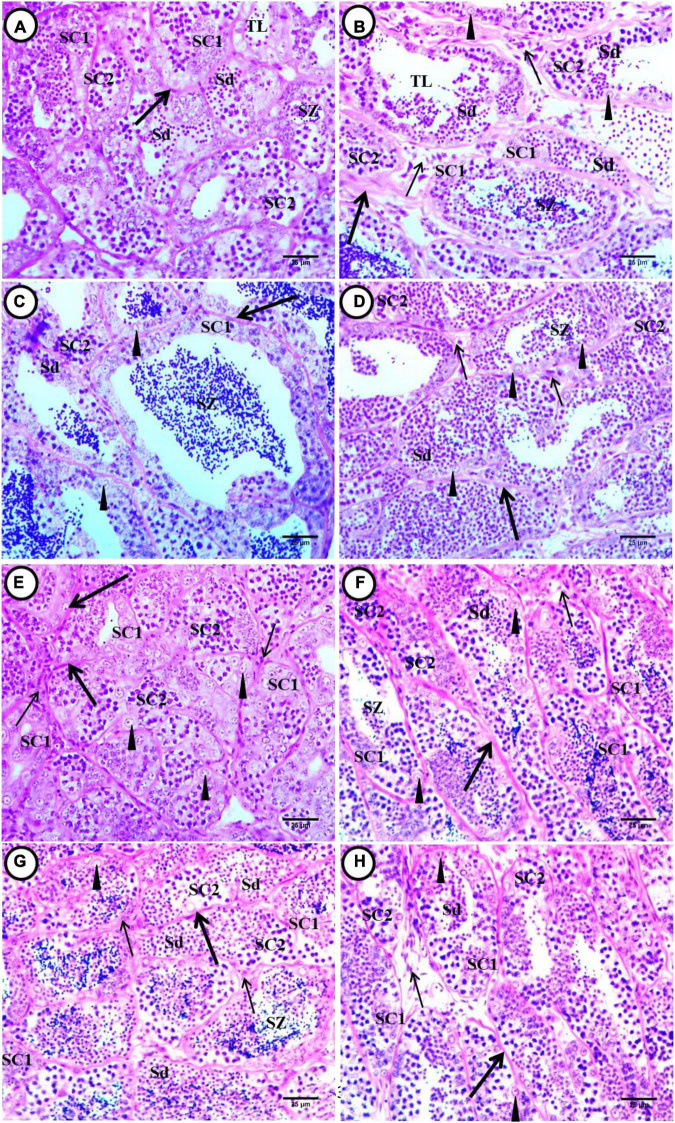
Transverse sections of juvenile African catfish (*C. gariepinus)* testes. **(A)** Control fish after exposure to 4-NP, **(B)** fish exposed to 0.1 mg/L 4-NP, **(C)** fish exposed to 0.2 mg/L 4-NP, **(D)** fish exposed to 0.3 mg/L 4-NP, **(E)** control fish after recovery period, **(F)** fish exposed to 0.1 mg/L 4-NP dose after the recovery period, **(G)** fish exposed to 0.2 mg/L 4-NP dose after the recovery period, and **(H)** fish exposed to 0.3 mg/L 4-NP dose after the recovery period. Seminiferous tubule contained primary spermatocytes (SC1), secondary spermatocytes (SC2), spermatids (Sd), spermatozoa (SZ), Sertoli cells (arrowhead), lumen tubule (TL), intertubular connective tissue (thick arrow), and interstitial Leydig cells (thin arrow). (H&E ×400).

The testes of *C. gariepinus* juvenile in the control group showed normal architecture of spermatogenic stages. The large cells were germ cells or spermatogonia and were located along the periphery of seminiferous tubules with Sertoli cells, with sharp boundaries and vesicular nucleus. There were primary spermatogonia (sperm mother cells) that were the largest cells of the germinative lineage, which could take place either in isolation or in groups within the cysts in the seminiferous tubules. They are large oval cells with very scarce, lightly eosinophilic cytoplasm and a large round nucleus with a single nucleolus. A cluster of secondary spermatogonia enclosed within a cyst is formed by a sequence of recurrent mitotic divisions in each initial spermatogonium. Large primary spermatocytes were present in groups surrounded by fine connective tissues, and each group contains various primary spermatocytes that were smaller in size than secondary spermatogonia; hematoxylin stains the nucleus heavily, while dyes have limited affinity for the cytoplasm. Secondary spermatocytes were somewhat smaller than primary spermatocytes with deeply stained nuclei. Spermatids were small with scant cytoplasm and with strongly condensed basophilic nucleus. Spermatids inside the cyst increased in number toward the center of the testes. The cyst ruptures at this point, releasing the spermatids into the testis lumen, where they mature. Interstitial cells (Leydig cells) were noticed in the supporting fibrous connective tissues and form groups near the blood capillaries in between seminiferous tubules (Figures 2A,E).

In fish exposed to 0.1 mg/L 4-NP, testes showed enlargement of seminiferous tubules surrounded by thick, dense, wavy, and irregular interlobular connective tissues; each tubule contains patches of different spermatogenic stages (primary and secondary spermatocytes, spermatids, and sperm), which were located in the tubule lumen. The enhancement of maturation was noticed by the transformation of spermatids into sperms, and after spawning, the lumen of seminiferous tubules contained degenerated spermatids and sperms. Detachment of a few spermatids and atrophy of Sertoli cells were observed. There was a widening of intertubular connective tissues and an increase in Leydig cells. Congested blood vessels were also noticed in intertubular connective tissues near Leydig cells (Figure 2B).

In fish exposed to 0.2 and 0.3 mg/L 4-NP, we observed pathological conditions such as thinning of intertubular connective tissues when compared with 0.1 mg/L 4-NP, which appeared degenerated and less acidophilic in staining. Loss of regular morphology of spermatogenic stages and degenerated ones were observed. Widening of intertubular connective tissues and vacuolation and degeneration of Leydig cells were noticed. Fusion and deformation in the shape of seminiferous tubules were observed. Detachment and degeneration of spermatids and sperms and atrophy of Sertoli cells were observed. The dominant stages were a few hazy staining primary spermatocytes, secondary spermatocytes, spermatids, and sperms (Figures 2C,D). In fish exposed to 0.3 mg/L 4-NP, the lumen of most tubules appeared devoid or contained few sperms, and most of the cells were in spermatid stages located in the center of tubules (Figure 2D). While in fish exposed to 0.2 mg/L 4-NP, there was an increase in sperm production (Figure 2C).

After post-exposure, the fish exposed to 0.1 mg/L 4-NP showed thinning of peritubular connective tissues, narrowing of intertubular space, deformation, and fusion of seminiferous tubules. The main stages were the secondary spermatocytes, followed by spermatids and a few stages of primary spermatocytes. The lumen of tubules contains few sperms. We also observed other pathological conditions such as necrosis and vacuolation in the basal cells’ primary spermatocytes and degeneration of interstitial Leydig cells (Figure 2F). In fish exposed to 0.2 mg/L 4-NP, there were shrunken seminiferous tubules, and the restricted stages of spermatogenesis contain few cells. Thickening of intertubular connective tissues was observed. Fusion of seminiferous tubules and vacuolation of basal cells were noticed. Decrease and degeneration of Leydig cells and lumens contain stages of spermatids and sperm. The main stages were the spermatids, degenerated and vacuolated primary spermatocytes, secondary spermatocytes, and a few sperms (Figure 2G). In fish exposed to 0.3 mg/L 4-NP, there was deformation and fusion in seminiferous tubules shapes, which were surrounded by wavy and discontinuous bundles of connective tissues. There was a widening of intertubular space, which contains degenerated and vacuolated interstitial Leydig cells. The lumen contained different stages of detached spermatids and few sperms. The main stages were the secondary spermatocytes followed by spermatid and few sperms. There was a displacement of vacuolated primary spermatocytes from the basement membrane (Figure 2H).

### Artesia Percentage and Sertoli Cells Numbers

The ovary Artesia and the number of Sertoli cells per seminiferous tubules after exposure to 4-NP for 15 days were recorded in Table 3. Significant variances found between treatment groups and the control (*P* < 0.05) were evident in both ovary Artesia and the number of Sertoli cells. The ovary Artesia exhibiting the significance of *P* < 0.05 between treatments tend to increase with the increase of 4-NP doses from 0.0 in the control to 0.3 mg/L, while several Sertoli cells exhibit a significant decrease with such increased doses. Similar patterns of significance and trend variations toward increase or decrease were recorded post-exposure for 15 days (Table 3). There was a slight decrease in ovary Artesia between treatments when compared with the exposure period and a slight increase in the number of Sertoli cells when compared with the exposure period.

**TABLE 3:** Quantitative histological analysis of ovary Artesia and Sertoli cells numbers [mean ± SE (range)] in juvenile African catfish (*C. gariepinus*) exposed to 0.1, 0.2, and 0.3 mg/L4-NP for 15 days and recovery for 15 days.

Treatments parameters	Control		4-NP As (0.1 mg/L)		4-NP As (0.2 mg/L)		4-NP As (0.3 mg/L)	
	Exposure	Recovery	Exposure	Recovery	Exposure	Recovery	Exposure	Recovery
Ovary% artesia	1.32 ± 0.28^a^ (0–4)	1.05 ± 0.22^a^ (0–4)	5.1 ± 0.29^b^ (3–8)	3.05 ± 0.27^b^ (1–5)	6.1 ± 0.34^b^ (4–9)	5.47 ± 0.23^c^ (4–7)	13.55 ± 0.6^c^ (10–18)	8.9 ± 0.25^d^ (7–11)
Sertoli cells numbers%	27.05 ± 0.95^a^ (19–35)	26 ± 0.84^a^ (19–31)	13.95 ± 0.41^b^ (11–17)	14.75 ± 0.34^b^ (12–18)	12.3 ± 0.33^b^ (10–15)	13.05 ± 0.32^bc^ (11–15)	10.5 ± 0.32^c^ (8–13)	11.65 ± 0.36^c^ (9–15)

*Significant differences from the control group values were accepted at P < 0.05. n = 3. Values with the same letters within a parameter are not significantly different at 0.05 level (horizontal comparison).*

### Detection of Apoptosis in Erythrocytes and Brain Tissue (Telencephalon) and 4-NP Residues in the Brain

Supplementary Figures 1, 2 show typical acridine orange-stained erythrocytes and apoptotic brain cells, which appeared light green under the microscope in samples from all four groups. A higher frequency of apoptotic brain cells (circled in red) than non-apoptotic cells (yellow) were observed in the 4-NP groups compared to the control. Similar patterns of apoptosis were detected after the recovery period. The apoptotic brain cells were found to be associated with increased 4-NP doses due to the 4-NP residues found in the brain: 17.67 ± 3.84*^a^* (10–22), 66.33 ± 4.01*^b^* (60–74), and 135.67 ± 11.92*^c^* (112–150) μg/g for the 0.1, 0.2, and 0.3 mg/L treatments, respectively, after the exposure period and 0 ± 0*^a^* (0–0), 13.33 ± 13.33*^a^* (0–40), and 51 ± 9.71*^b^* (38–70) μg/g for the 0.1, 0.2, and 0.3 mg/L treatments, respectively, after the recovery period.

### Percentages of Erythrocyte Malformations and Apoptosis

**FIGURE 3:**
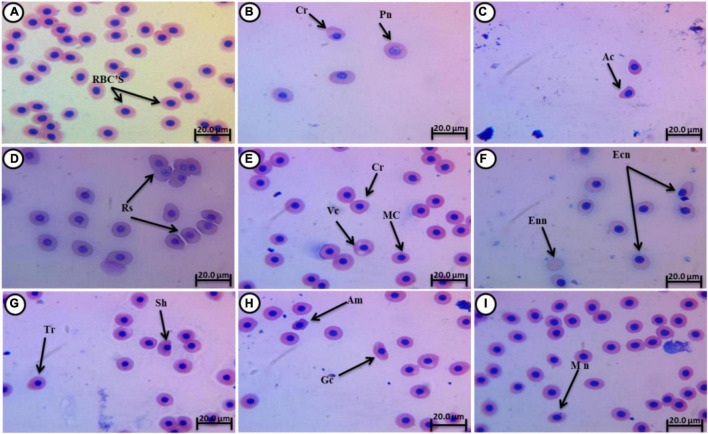
Giemsa-stained blood smears from juvenile C. *gariepinus*, showing normal erythrocytes and **(A)** deformed erythrocytes **(B–I)** after exposure to 4-NP for 15 days. Tr, teardrop cell; Cr, crenated cell; Pn, pale nucleated cell; Rs, Redoulex-shaped cell; Ac, acanthocyte; Vc, vacuolated cell; Ecn, eccentric nucleus; Mn, micronucleated cell; Enn, enucleated cell; Am, amoeboid cell; Mac, macronucleated cell; Gc, genuine cell; and Sk, sickle cell. (Magnification = 1,000×).

Normal erythrocytes of juvenile *C. gariepinus* are rounded with a centrally located nucleus (Figure 3A). Morphological deformations of erythrocytes were recorded in the experimental groups exposed to fatal concentrations of 4-NP (0.1, 0.2, and 0.3 mg/L). These deformations (Figures 3B–I) included crenated cells (Cr), acanthocytes (Ac), teardrop cells (Tr), sickle cells (Sk), micronucleated cells (Mn), pale nucleated cells (Pn), Redoulex-shaped cells (Rs), vacuolated cells (Vc), enucleated cells (Enn), amoeboid cells (Am), eccentric nucleus (Ecn), macronucleated cells (Mac), and genuine cells (Gc).

Table 4 shows the percentages of erythrocyte alterations, nuclear abnormalities, and apoptosis after 15 days of 4-NP exposure and after 15 days of recovery. Significant differences (*P* < 0.01) between the treatment groups and the control group were recorded in all erythrocyte parameters except in Table 4. After the recovery period, significant variations were still evident between the groups in all erythrocyte parameters except for Sk and Rs (Table 3), indicating that the effects of 4-NP were lost beyond exposure. In many parameters, insignificant differences were evident among the 4-NP groups, but each of these groups differed significantly from the control after the exposure period (Vc, Ac, Ecn, Pn, Sc, Rs, and Cr) and the recovery period (Vc, Ac, Sc, and Cr). These differences in erythrocyte parameters increased with 4-NP concentration, suggesting that the effects of 4-NP on erythrocyte deformations and apoptosis are dose-dependent (Table 4).

**TABLE 4:** Erythrocytes morphological alterations, nuclear abnormalities, and apoptosis (mean ± SE) in juvenile African catfish (*Clarias gariepinus)* exposed to 0.1, 0.2, and 0.3 mg/L 4-NP for 15 days and recovery for 15 days (*n* = 3).

Treatment parameters	Control		0.1 mg/L 4-NP		0.2 mg/L 4-NP		0.3 mg/L 4-NP	
	Recovery	Exposure	Recovery	Exposure	Recovery	Exposure	Recovery	Exposure
Vc	0.7333 ± 0.158^a^ (0–2)	0.46 ± 0.178^a^ (0–4)	3.83 ± 0.38^b^ (0–9)	3.33 ± 0.61^b^ (0–12)	4.23 ± 0.35^b^ (1–10)	3.7 ± 0.37^b^ (0–8)	4.5 ± 0.33^b^ (1–10)	3.86 ± 0.51^b^ (0–10)
Ac	0.23 ± 0.092^a^ (0–2)	0.233 ± 0.104^a^ (0–2)	4.3 ± 0.35^b^ (1–10)	2.86 ± 0.38^b^ (0–7)	4.43 ± 0.34^b^ (2–10)	3.2 ± 0.29^b^ (0–7)	4.8 ± 0.37^b^ (0–10)	3.3 ± 0.35^b^ (0–7)
Non	0.7 ± 0.16^a^ (0–3)	0.166 ± 0.08^a^ (0–2)	0.76 ± 0.189^a^ (0–3)	0.3 ± 0.108^a^ (0–2)	0.866 ± 0.177^a^ (0–3)	0.66 ± 0.18^ab^ (0–3)	1.4 ± 0.265^a^ (0–4)	1.13 ± 0.27^b^ (0–5)
Sk	1.33 ± 0.236^a^ (0–4)	1.33 ± 0.24^a^ (0–4)	2.6 ± 0.294^b^ (0–6)	1.63 ± 0.29^a^ (0–5)	3.46 ± 0.28^bc^ (0–7)	1.73 ± 0.31^a^ (0–6)	4.03 ± 0.319^c^ (0–8)	1.96 ± 0.25^a^ (0–4)
Ecn	3.5 ± 0.28^a^ (0–7)	1.63 ± 0.35^a^ (0–7)	4.03 ± 0.32^ab^ (0–8)	3.7 ± 0.45^bc^ (0–9)	4.93 ± 0.47^b^ (0–10)	3.13 ± 0.423^b^ (0–8)	5.2 ± 0.52^b^ (0–12)	4.53 ± 0.24^c^ (2–8)
Pn	0.366 ± 0.112^a^ (0–2)	0.766 ± 0.189^a^ (0–3)	1.366 ± 0.216^b^ (0–3)	1.36 ± 0.28^ab^ (0–4)	1.63 ± 0.301^b^ (0–6)	1.36 ± 0.305^ab^ (0–6)	2.2 ± 0.375^b^ (0–9)	1.7 ± 0.3^b^ (0–5)
Sc	2.2 ± 0.37^a^ (0–9)	1.7 ± 0.3^a^ (0–5)	3.7 ± 0.267^b^ (2–7)	3 ± 0.38^b^ (0–7)	3.86 ± 0.444^b^ (0–8)	3.2 ± 0.29^b^ (0–7)	4.5 ± 0.44^b^ (0–10)	3.43 ± 0.35^b^ (0–8)
Tr	0.73 ± 0.16^a^ (0–3)	0.47 ± 0.18^a^ (0–4)	2.96 ± 0.36^b^ (0–7)	2.1 ± 0.32^b^ (0–5)	3.7 ± 0.38^b^ (0–8)	3.2 ± 0.32^bc^ (0–7)	5.2 ± 0.517^c^ (0–12)	4.3 ± 0.47^c^ (0–10)
Mac	1.5 ± 0.25^a^ (0–5)	1.36 ± 0.31^a^ (0–6)	2.2 ± 0.408^a^ (0–7)	1.63 ± 0.35^a^ (0–7)	2.4 ± 0.32^a^ (0–6)	2.23 ± 0.302^ab^ (0–6)	3.7 ± 0.38^b^ (0–8)	3.13 ± 0.42^b^ (0–8)
Rs	0.167 ± 0.069^a^ (0–1)	0.166 ± 0.08^a^ (0–2)	0.37 ± 0.112^ab^ (0–2)	0.23 ± 0.104^a^ (0–2)	0.37 ± 0.139^ab^ (0–3)	0.23 ± 0.09^a^ (0–2)	0.8 ± 0.19^b^ (0–3)	0.466 ± 0.18^a^ (0–4)
Enn	0.17 ± 0.08^a^ (0–2)	0.7 ± 0.186^a^ (0–4)	1.7 ± 0.23^b^ (0–4)	1.13 ± 0.22^ab^ (0–4)	2.13 ± 0.28^b^ (0–5)	1.6 ± 0.26^bc^ (0–4)	3.46 ± 0.28^c^ (0–7)	2.1 ± 0.32^c^ (0–5)
Cr	2.2 ± 0.408^a^ (0–7)	1.7 ± 0.31^a^ (0–6)	4.2 ± 0.49^b^ (0–10)	3.8 ± 0.38^b^ (0–9)	4.4 ± 0.47^b^ (0–10)	4.3 ± 0.48^b^ (0–10)	4.8 ± 0.39^b^ (0–9)	4.5 ± 0.24^b^ (2–8)
**Apoptosis%**	2.43 ± 0.23^a^ (0–5)	2.3 ± 0.205^a^ (0–4)	5.23 ± 0.324^b^ (3–11)	2.4 ± 0.148^a^ (1–4)	8.56 ± 0.485^c^ (1–15)	4.17 ± 0.225^b^ (2–8)	15.03 ± 0.646^d^ (8–22)	9.63 ± 0.33^c^ (4–12)

*Significant differences from the control group values were accepted at P < 0.05. Values with the same letters within a parameter are not significantly different at 0.05 level (horizontal comparison). Vc, vacuolated cell; Ac, acanthocyte; Non, notched nucleated cell; Sk, sickle cell; Ecn, eccentric nucleus; Pn, pale nucleated cell; Sc, swelled cell; Tr, tear-drop cell; Mac, macronucleated cell; Rs, Redoulex shape cell; Enn, enucleated cell and Cr, crenated cell.*

## Discussion

This study stated a significant decrease in plasma FSH levels in juvenile African catfish *(Clarias gariepinus)* exposed to 0.1, 0.2, and 0.3 mg/L 4-NP for 15 days. These findings were in accordance with other studies (Shirdel et al., 2020) that indicated that E2 could inhibit the synthesis and secretion of FSH in salmonids. The 4-NP effect on FSH response revealed a different trend where it is impaired (Maeng et al., 2005) or no effect is recorded (Yadetie and Male, 2002). Also, the results of the current study reported a significant increase in LH levels in juvenile African catfish *(Clarias gariepinus)* exposed to 0.1, 0.2, and 0.3 mg/L 4-NP for 15 days. These findings were in accordance with results verified in African catfish exposed to 4-NP (Sayed et al., 2012a,b) and with results of Shirdel et al. (2020) in non-ylphenol-exposed Caspian trout smolts. The study of organ distribution using (3H)-NP indicated that non-ylphenol passed the blood-brain barrier (BBB; Arukwe et al., 2000). Yadetie and Male (2002) suggested that NP mimics E2 *via* acting on both pituitary and brain levels. Non-ylphenol could induce the LH levels through the mechanism of action of 4-NP on estrogen receptors in the LH expressing cells in the pituitary (Yadetie and Male, 2002). In contrast to FSH, the pituitary LH level is regulated through a positive feedback action by plasma estradiol (Dickey and Swanson, 1999). Thus, the elevation of plasma LH level in juvenile African catfish (*C. gariepinus*) exposed to 0.1, 0.2, and 0.3 mg/L 4-NP may be due to the positive feedback effects caused by the elevated level of plasma estradiol as a result of non-ylphenol exposure as explained by Shirdel et al. (2020) in non-ylphenol-exposed Caspian trout smolts.

The present study reported a significant decrease in plasma T levels. These data corroborated by previous reports of 4-NP in other fish (Lavado et al., 2004; Shirdel et al., 2020), the male *C. gariepinus*, and male *Heteropneustes fossilis*, after exposure to a gamma-hexachlorocyclohexane pesticide, which decreases T levels. Similarly, in Carp *(Cyprinus carpio)* collected from wastewater treatment plants (WWTP) polluted by NP and NPEs, the T levels diminished. In Caspian trout smolts exposed to 4-non-ylphenol, Han et al. (2004) thought that the inhibitory effect of non-ylphenol on P450c17, the enzyme functioning in the testosterone biosynthesis, resulted in inhibition of serum testosterone in male rats after non-ylphenol exposure.

Shirdel et al. (2020) reported that histopathological alterations resulting from non-ylphenol exposure have not been widely studied, however, non-ylphenol has been shown to cause degeneration, vacuolation, and necrosis in gonads of exposed fish. These impacts and alterations, in agreement with previous studies, reported degeneration, necrosis, and vacuolation, such as those indicated in testicular structures (Amaninejad et al., 2018). Gautam (2011) demonstrated that a lower concentration of 4-NP can ovulate and mature oocytes through stimulation of sexual glands’ preparatory phase in female catfish *H. fossilis*. However, at the higher concentrations, a delay in the growth of sexual glands and maturation, in addition to a significant increase in atresia oocytes of stages 1 and 2, can also occur (Sayed and Ismail, 2017), which is indicated in the present study as a dose-dependent damage. A female fish also presented alterations in gonads tissue after exposure to 16 m/l of NP, and it also induced changes in the gonads of *Oreochromis niloticus*, especially in females (Rivero et al., 2008). The results of the present study indicated oocytes atresia and karyoplasmic clumping. According to Eni et al. (2019), alteration in fecundity by limiting the number of eggs produced may be a result of the presence of atretic oocytes in exposed fish. The results of the present study also indicated the presence of melanomacrophage in fish ovary exposed to 0.3 mg/L 4-NP, which may be one of the defense mechanisms to such estrogenic compound because of the increase of atretic follicles.

In this study, there were changes that led to abnormal development of testes, and this is consistent with the results of Harris et al. (2001), who reported that, after 100 mg NP/L exposure, gonadal development ceases and steroidogenesis was markedly inhibited. Duan et al. (2016) reported spermatogenic degeneration and pronounced deficits in epididymal sperm count, motility, and function after exposure of male rats to non-ylphenol (Kim et al., 2019). Recently, Adeogun et al. (2018) reported degenerations in the seminiferous tubules after exposure to phthalate esters (PEs) in *C. gariepinus* and Shirdel et al. (2020) reported spermatogenic cell degeneration and decrease in thickness of the lobule walls after exposure of Caspian brown trout (*Salmo trutta caspius*) smolts to non-ylphenol. Zade et al. (2018) reported vacuolation, alterations in germ cell structure such as sickle-shaped germ cells, and degeneration of the germ cells after exposure of *C. gariepinus to* non-ylphenol. A possible explanation for the above changes in the testis structure is the effect on the structure and number of Sertoli cells. The Sertoli cells play a major role in testicular tissue, including spermatozeugmata morphology regulation of spermatogenesis and spermiation, cysts formation, in which spermatogenesis takes place, support and nutrition of germ cells, residual bodies’ phagocytosis, and inhibin secretion.

Although the mechanism where estrogens and estrogenic chemicals, such as non-ylphenol, cause inhibitory or degenerative effects on testicular development and structure is still unknown, Zade et al. (2018) demonstrated that non-ylphenol acts on Sertoli cell and it may be responsible for disturbing its functions. Direct impacts on the testis can be cytotoxic, where the disruption is produced by destruction to the testis cells as a whole, or on the endocrine, where the functioning of specific cells, such as Sertoli cells, is disrupted due to endocrine failure (Kime, 1999).

The 4-NP is toxic at concentrations of 17–3,000 μg/L, depending on the species of fish (Mekkawy et al., 2011; Abou Khalil et al., 2017; Kotb et al., 2018; Sayed et al., 2019a). A dose-dependent damage caused by 4-NP was evident in the current study, with similar results having been reported in other works as well (Mekkawy et al., 2011; Al-Sharif, 2012; Sharma and Chadha, 2017). Carbosulfan (Nwani et al., 2011), 2,4-dicholorophenoxyacetic acid (Ateeq et al., 2005), UVA (Sayed et al., 2013, 2016c), and arsenic (Sayed et al., 2015) have also been found to cause damage in a dose-dependent manner. Different studies implied the variability in the effects of pollutants on hematological and biochemical indicators, such as RBCs, Hb, Hct, MCH, MCHC, MCV, and WBCs.

The significant reduction in WBC count due to increased 4-NP concentration indicates that these juvenile catfish are at a high risk of infection. Similar results were reported after exposure to diazinon (Adedeji et al., 2009). Shaluei et al. (2013) and Imani et al. (2015) observed an increase in the WBCs of silver-nanoparticle-exposed silverfish and rainbow trout, respectively. Hence, the changes in WBC count can be used as evidence for decreased immunity after exposure to toxic substances (Adedeji et al., 2009).

In this study, the 4-NP induced significant erythrocyte deformation and apoptosis in a dose-dependent manner, which was consistent with the findings of other studies on different pollutants, such as carbosulfan, 2,4-dicholorophenoxy acetic acid, UVA, and arsenic (Mekkawy et al., 2011; Al-Sharif, 2012; Sayed et al., 2013, 2015, 2016c; Sharma and Chadha, 2017; Osman et al., 2019). Morphological and nuclear irregularities in erythrocytes and micronuclei have been scored as biomarkers for genotoxicity after exposure to radiation and chemical pollution (Mekkawy et al., 2011, 2019; Sayed et al., 2013, 2016b; Sharma et al., 2014; Gautam et al., 2015; Sayed and Moneeb, 2015; Sayed and Mitani, 2017). An apoptosis induction after 4-NP treatment has been indicated in tissues and blood cells of many fish species (Murakawa et al., 2001; Mekkawy et al., 2011; Sayed and Hamed, 2017). Udroiu (2006) suggested that the high levels of erythrocytic damage and apoptosis seen in fish erythrocytosis are due to their short lifespan (1–3 months).

According to Kotb et al. (2018) and Sayed et al. (2019a), the toxic effects of 4-NP exposure may arise from different mechanisms, although this requires further research. The apoptotic impacts on erythrocytes, and the brain cells are due to DNA breakage. In this study, there was a proportional relationship between 4-NP residues in the brain and neural apoptosis. The 4-NP succeeded in transgressing the BBB, which is not a simple rigid or inert barrier (Banks, 1999). The BBB serves as a microenvironment to maintain brain homeostasis and protect it against toxic agents (Cserr and Bundgaard, 1984). Toimela and Tahti (2004) demonstrated that apoptosis is a common mechanism of neuronal cell death in response to pathological disturbances. It is worth mentioning that 4-NP represses glutathione peroxidase expression and inhibits the protective mechanisms of the cell (Yokel, 2002) despite tight junctions in the endothelial cells, which function as a seal to guard the brain (Abbott et al., 2010).

To our knowledge, this is the first research that investigated the recovery period (post-exposure) effect on the toxic effects caused by the 4-NP, where most of the biomarkers used were restored to normal or semi-normal status. In this regard, we can suppose that the effect of the 4-NP on the reproductive system of the African catfish is toxic and estrogenic more than just estrogenic.

## Conclusion

The release of this commercially important species into contaminated rivers with 4-non-ylphenol should be avoided since it can have serious consequences for its development and reproductive function. Our results also indicated that 4-NP caused cytotoxic effects, such as erythrocyte alterations and apoptosis, in a dose-dependent manner, and the recovery period greatly decreased the alteration level.

## Data Availability Statement

The raw data supporting the conclusions of this article will be made available by the authors, without undue reservation.

## Ethics Statement

The animal study was reviewed and approved by the Assiut University Ethical Committee.

## Author Contributions

AS and ZE: experimental design, methodology, data interpretation, and investigation. AS, ZE, UM, J-SL, and IM: writing and revision. All authors read and approved the final manuscript.

## Conflict of Interest

The authors declare that the research was conducted in the absence of any commercial or financial relationships that could be construed as a potential conflict of interest.

## Publisher’s Note

All claims expressed in this article are solely those of the authors and do not necessarily represent those of their affiliated organizations, or those of the publisher, the editors and the reviewers. Any product that may be evaluated in this article, or claim that may be made by its manufacturer, is not guaranteed or endorsed by the publisher.
