# Supplementary material for: Reproductive Toxicity and Recovery Associated With 4-Non-ylphenol Exposure in Juvenile African Catfish (Clarias garepinus)
Source: Front Physiol. 2022 Apr 11;13:851031. doi: 10.3389/fphys.2022.851031 (PMC9035889; doi:10.3389/fphys.2022.851031)
Supplement: Supplementary file 1 [file Data_Sheet_1.docx]

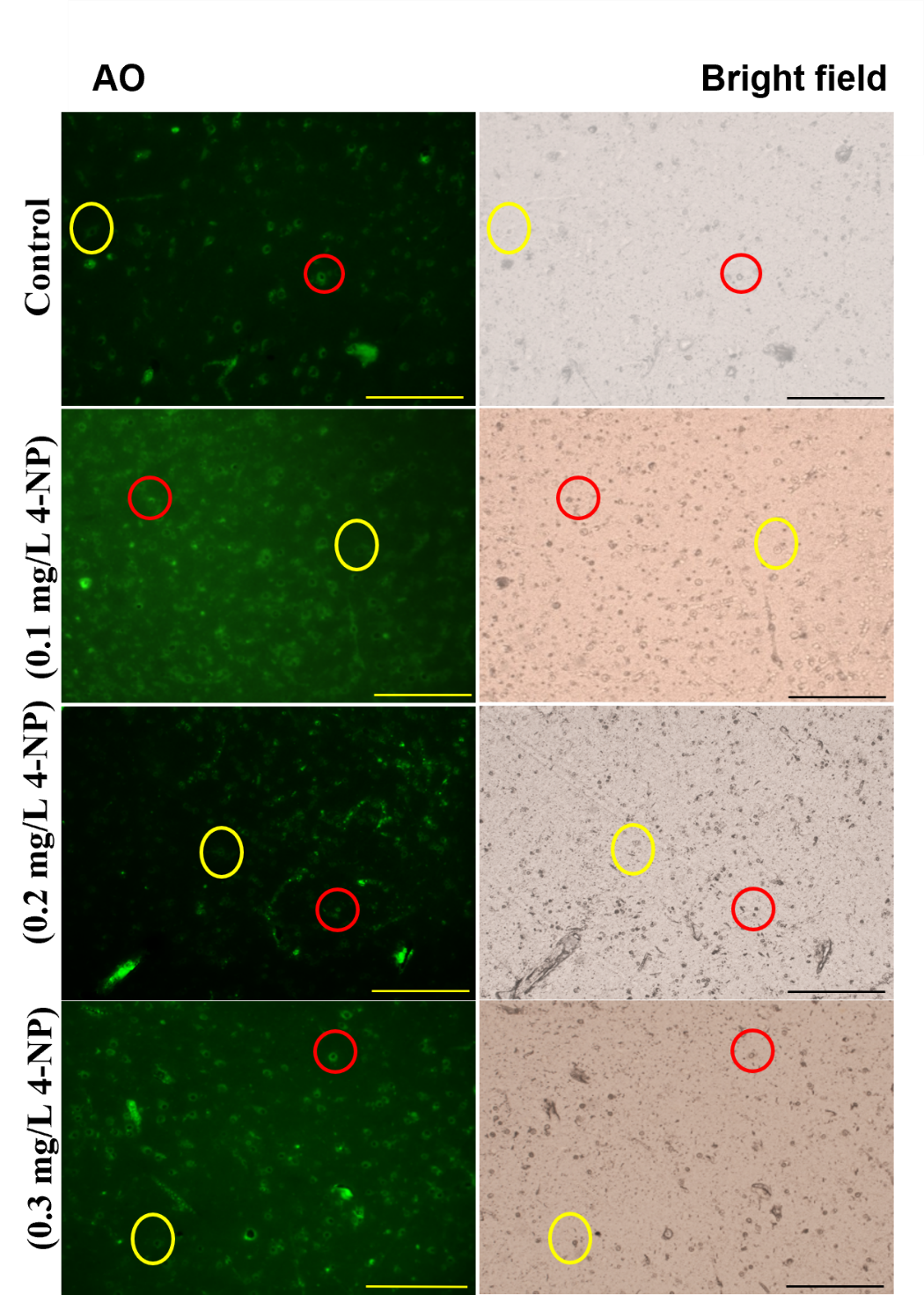


**Supplementary fig. 1**. Apoptosis detection in brain tissue of juvenile C. gariepinus exposed to 0.1, 0.2, and 0.3 mg/L 4-NP for 15 days. Cells fluorescing light green after acridine orange staining were considered apoptotic. Red circles indicate apoptotic cells, whereas yellow circles indicate non-apoptotic cells. (Scale bar = 50 μm).

**
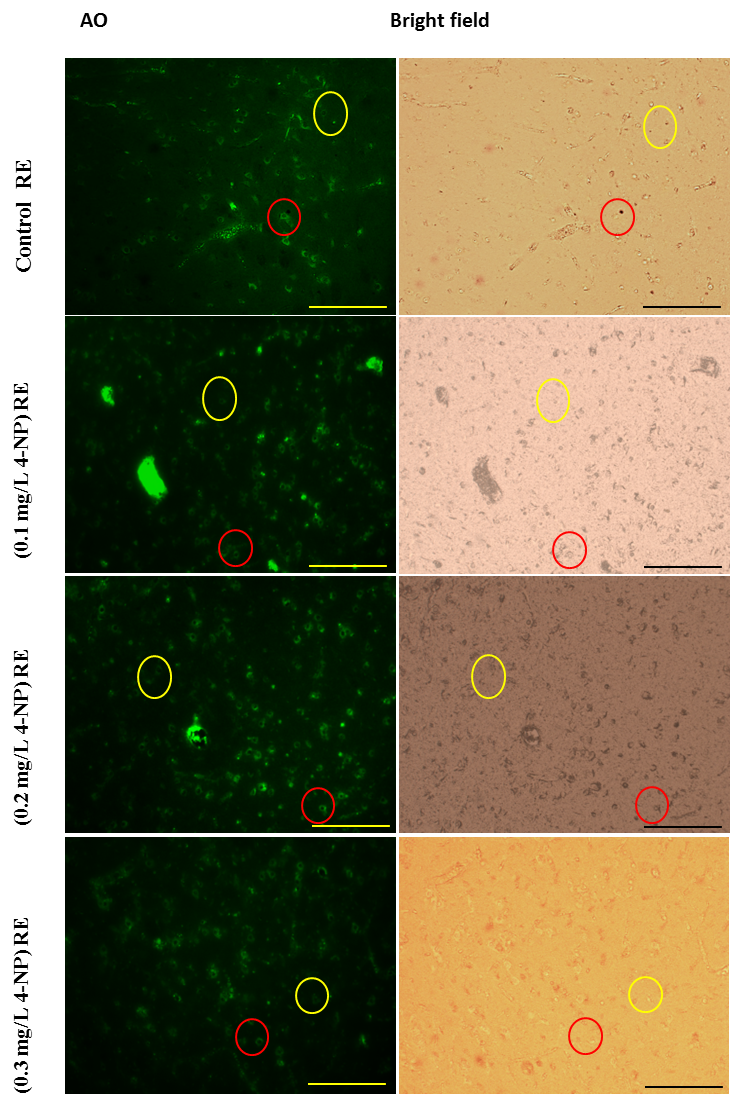
**

**Supplementary fig. 2**. Apoptosis detection in brain tissue of juvenile C. gariepinus exposed to 0.1, 0.2, and 0.3 mg/L 4-NP after a recovery period of 15 days. Cells fluorescing light green after acridine orange staining were considered apoptotic. Red circles indicate apoptotic cells, whereas yellow circles indicate non-apoptotic cells. (Scale bar = 50 μm).
